# Distributed transformer for high order epistasis detection in large-scale datasets

**DOI:** 10.1038/s41598-024-65317-5

**Published:** 2024-06-25

**Authors:** Miguel Graça, Ricardo Nobre, Leonel Sousa, Aleksandar Ilic

**Affiliations:** INESC-ID, Instituto Superior Técnico, 1000-029 Lisbon, Portugal

**Keywords:** Bioinformatics, Machine learning, High performance computing, Computational biology and bioinformatics, Hardware and infrastructure, Machine learning

## Abstract

Understanding the genetic basis of complex diseases is one of the most important challenges in current precision medicine. To this end, Genome-Wide Association Studies aim to correlate Single Nucleotide Polymorphisms (SNPs) to the presence or absence of certain traits. However, these studies do not consider interactions between several SNPs, known as epistasis, which explain most genetic diseases. Analyzing SNP combinations to detect epistasis is a major computational task, due to the enormous search space. A possible solution is to employ deep learning strategies for genomic prediction, but the lack of explainability derived from the black-box nature of neural networks is a challenge yet to be addressed. Herein, a novel, flexible, portable, and scalable framework for network interpretation based on transformers is proposed to tackle any-order epistasis. The results on various epistasis scenarios show that the proposed framework outperforms state-of-the-art methods for explainability, while being scalable to large datasets and portable to various deep learning accelerators. The proposed framework is validated on three WTCCC datasets, identifying SNPs related to genes known in the literature that have direct relationships with the studied diseases.

## Introduction

Advancements in DNA sequencing in the past 40 years have paved the way from analyzing small sequences to mapping the entire human genome. This technological breakthrough has allowed for the emergence of Genome-Wide Association Studies (GWAS)^1^, a research approach that aims to unveil correlations between complex diseases and Single Nucleotide Polymorphisms (SNPs), a common type of genetic variation. GWAS study a phenotype, a set of observable characteristics, such as a disease, and define the individuals of a population by the presence (case) or absence (control) of the studied traits. The rationale for this methodology lies in assuming that common diseases have common underlying influential genetic variants across a population^2^. Some examples of the success of GWAS include the association between the IL-12/IL-23 pathway and the development of Crohn’s Disease^3^, as well as the discovery of the PTPN22 gene’s influence in autoimmune diseases^4^.

The approach for GWAS makes a crucial assumption: SNPs are independently correlated to the studied phenotype. Therefore, SNPs can be tested individually for statistical relevance to the disease, while neglecting gene-environment and gene-gene interactions, known as the “missing heritability” problem in the literature^5^. The combinatorial effect that arises when two or more SNPs interact is known as epistasis and may play a fundamental role on the missing heritability problem. Research on epistasis has focused on the detection of SNP interactions to explain complex diseases, such as Late Onset Alzheimer’s Disease^6^.

Finding the optimal interacting SNP combination to explain a disease implies the exhaustive evaluation of all possible cases, which presents a current computational challenge. As an example, on WTCCC datasets, as many as 500,000 SNPs must be analyzed, resulting in 125 possible billion combinations of two genes (second order epistasis) and 20 quadrillion combinations of three genes (third order epistasis) to be analyzed. In these datasets, analyzing combinations of three of more SNPs (high order epistasis) results in an infeasible execution time for evaluation of all combinations, due to the exponential growth of epistatic combinations to evaluate. However, high order epistasis is of the utmost importance to understand genetic vulnerability to certain diseases and is a current important research topic in precision medicine^7^.

Research on this area has focused on accelerating epistasis detection by using parallel accelerators, such as CPUs, GPUs, and FPGAs. Efficient solutions for exhaustive search of second and third order epistasis^8,9^ have already been proposed in the literature. Frameworks for forth to sixth order also exist^10–13^, but these have only been tested on datasets with a few thousand SNPs, demonstrating that optimized exhaustive searches are not adequate for high order epistasis detection. For this reason, non-exhaustive methods, spanning from filtering to machine learning^14^, have been proposed as an alternative solution.

Studies demonstrate that Deep Neural Networks (DNNs) have a high potential to identify patterns between SNPs and the observed phenotype^15^, even for large-scale datasets. Furthermore, DNN-based approaches are agnostic to the search order, implying that epistatic interactions of any order can be detected, while keeping a constant training time. However, DNN-assisted works on epistasis detection suffer from two major drawbacks. First, deep learning algorithms are limited by computational demands. Accuracy is correlated with the number of parameters, but increasing the network’s size comes at the cost of increased memory and computation capacities and may lead to overfitting the model. Second, the predominant purpose of DNNs is to classify data, and while good accuracy can be achieved for genomic datasets, it is necessary to know which SNPs are interacting for true epistasis detection. The missing biological interpretation is essential for epistasis, which is far from being a trivial task (and most of the time impossible), since most DNNs are black-box models that are not directly interpretable. Only a few works in the literature have attempted to bridge the gap in interpreting epistasis using common DNN architectures, such as MLPs with Layerwise Relevance^16^, CNNs with saliency maps^17^, and, more recently, transformers with attention scores for interpretation^18^, having managed to detect SNP interactions up to fifth order. While transformers have shown the capability to outperform other DNN architectures in epistasis detection interpretation^18^, it is still unknown if they are suitable for finding SNP interactions in real, large-scale datasets, if even higher-order interactions can be found with these DNNs, and how well they scale in systems with distributed AI accelerators, such as super-computers.

This paper aims to address the limitations of the state-of-the-art by proposing a framework based on a transformer model that partitions the key matrix (corresponding to the key-vector layer of transformers) in subsets and is trained on subset combinations to identify relevant SNPs for epistasis on large-scale datasets through various network interpretation metrics, namely attention scores and gradient calculation. The framework is tested on simulated datasets covering a broad variety of epistasis scenarios, outperforming other interpretation-based machine learning models up to eighth order interactions, which, to the best of our knowledge, has never been done in the literature. A scalability analysis is also performed on a wide range of AI hardware platforms. Finally, the proposed model is evaluated on three WTCCC datasets, regarding Coronary Artery Disease (CAD), Rheumatoid Arthritis (RA), and Inflammatory Bowel Disease (IBD), where some of the reported SNPs are related to genes that have been shown in state-of-the-art works to have individual and epistatic correlations with the aforementioned diseases, demonstrating its reliability for epistasis detection.

## Results

### A transformer neural network for high order epistasis detection

The aim of this study is to develop a deep learning model that is capable of detecting high order epistasis in real GWAS datasets. A transformer-based model is proposed, based on an encoder-only architecture, where, in the attention module, the key vector is split into multiple partitions (SNP subsets). Subset combinations are generated for multiple attention calculations within the model to obtain, for each SNP, multiple attention scores that are summed together. Furthermore, after training, gradients from the output label to the encoder’s output can be calculated for each SNP, providing a more robust network interpretation.

To cover a broad variety of epistasis models, synthetic datasets are generated according to an epistasis model (additive, multiplicative, threshold, and xor)^19^, Minor Allele Frequency (MAF) (0.05, 0.1, 0.2, 0.5), heritability (0.01, 0.05, 0.2, 0.4), and interaction order (second to eighth order). For each combination of these four parameters, 100 datasets are generated, with the exception of combinations related to the multiplicative model, which, due to its complexity^20^, allows only for second to fifth order interactions, considering the chosen values for MAF and heritability. All datasets are balanced (i.e., equal number of cases and controls), with 800 controls, 800 cases and a number of SNPs set to 1000.

To maximize the proposed model’s performance, it is necessary to search for optimal parameters. Regarding the transformer’s encoder block, the hyperparameters from another work in the literature^18^ are employed (spectral embedding as a strategy for positional embeddings, hyperbolic tangent as the activation function, and enforce the weight matrices in the attention layer to have 90% zero values). For the proposed framework, additional hyperparameters need to be considered, namely the number of partitions for the key vector, the combination size for the partitions, and the interpretation strategy to use.

The results of this hyperparameter search are shown in Table S1 in the Supplementary Material. The main takeaway is that dividing the key vector in 6 partitions with combinations of size 2 provides the best detection power when scaled element-wise sum of gradients and attention scores are used. Therefore, these are the optimal hyperparameters for the following experiments to tackle high order epistasis detection.

### State-of-the-art comparison

To compare the detection power of the proposed framework, a work based in MLPs^16^, referred herein as DeepCOMBI, as well as works based on CNNs^17^ and transformers^18^, are used. DeepCOMBI relies on a MLP to predict phenotypes and Layerwise Relevance Propagation (LRP) for interpretation and detection of SNP interactions; the CNN approach^17^ creates saliency maps for SNP interpretation; the transformer-based framework^18^ uses only attention scores to identify relevant SNPs without further modifications to the network’s architecture or other interpretation metrics. In addition to detection power, recall, precision, and F1-score are also calculated (see Table S2 in Supplementary Material) according to the procedure described in MACOED^21^.

**Figure 1:**
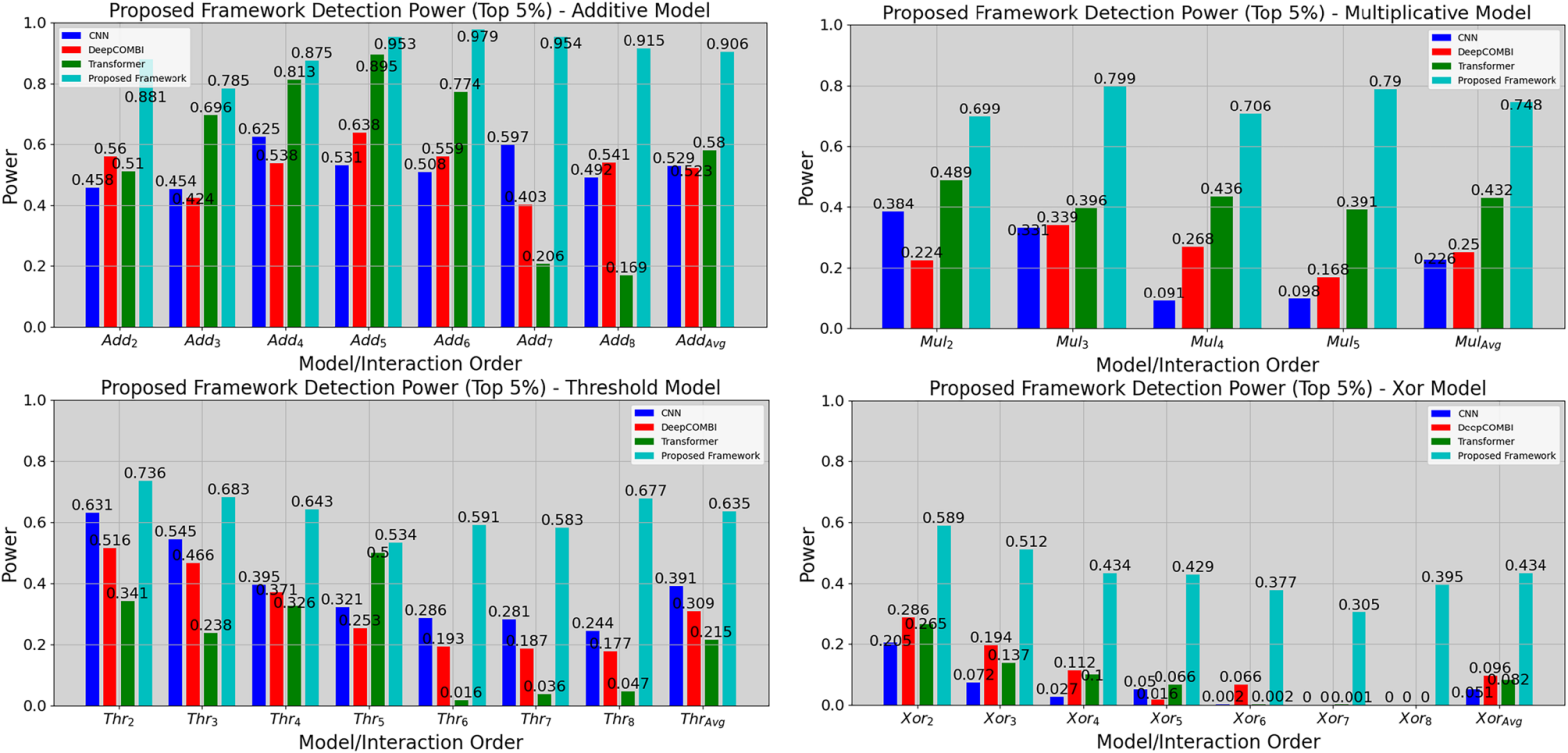
Detection power evaluated on the top 5% best SNPs for various interpretation-based neural networks in Additive (left-top), Multiplicative (right-top), Threshold (left-bottom) and Xor (right-bottom) epistasis models.

Figure 1 presents the detection power, for each network, on the additive, multiplicative, threshold, and xor models, across various interaction orders. This detection power is calculated according to each network’s capability to identify the interacting SNPs within the top 5%, according to their metric (e.g., in the case of the proposed framework, for each SNP, its sum of attention score and gradient is calculated and, after sorting the values, the interacting SNPs should be in the top 5% to consider the interaction as being detected). For each interaction order, 1600 datasets are evaluated (as there are 16 combinations of $h^2$ and MAF, and for each, 100 simulated datasets are created), the only exception being for multiplicative fifth order interactions, where only 1000 datasets were created, as some combinations of $h^2$ and MAF are impossible for fifth order, due to the multiplicative model’s complexity^20^. The reported detection power for each interaction order represents, for each network, the percentage of datasets in which all interacting SNPs are found in the top 5%.

On average, the proposed framework’s detection power for additive, multiplicative, threshold, and xor models is 90.6%, 74.8%, 63.5%, and 43.4%, respectively. For comparison, the second best detection power, on average, for each model, is 58% (transformer with attention scores only), 43.7% (transformer with attention scores only), 39.1% (CNN) and 9.6% (DeepCOMBI). The proposed approach also outperforms other DNN-based approaches in terms of recall, precision, and F1-score, achieving average increases of 30.6%, 26.9%, and 33.3% for recall, precision, and F1-score, respectively, when compared to the second best method (see Table S2 in Supplementary Material).

### Scalability analysis on AI hardware

#### Dataset scalability

To investigate the performance achieved with the proposed method and the scalability with the input datasets, experiments on different hardware architectures and devices are devised. Because the proposed transformer-based approach is agnostic to interaction orders, as opposed to exhaustive methods, and no assumptions are made on the input dataset, the model’s training time remains constant, as long as the architecture and number of epochs is fixed. In these circumstances, the only parameters that can influence the transformer’s running time are the number of patients (*P*) and the number of SNPs (*N*).

Synthetic datasets with a SNP range between 256 and 16,384 and a patient range between 1024 and 16,384 are generated to target five AI accelerators: Graphcore IPU GC-200^22^, Google TPU V4^23^, Intel PVC^24^, NVIDIA A100^25^, and the AMD MI250X LUMI supercomputer^26^.

**Figure 2:**
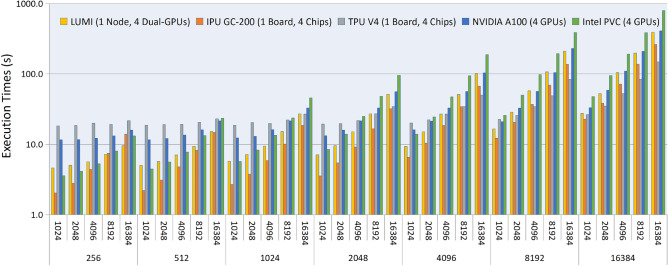
Average transformer training time for various dataset sizes on LUMI, NVIDIA A100, Intel PVC, Graphcore IPU GC-200, and Google TPU V4, from 1024 to 16,384 patients and from 256 to 16,384 SNPs.

For each combination of SNPs and patients, 100 balanced datasets are generated to train the proposed transformer model on each device and measure the average training time. For LUMI, this analysis is done on a single node with 4 dual GPUs; for NVIDIA A100 and Intel PVC, a set of 4 GPUS is used for training; for Graphcore IPU GC-200 and Google TPU V4, the training is done on individual boards with 4 chips. Figure 2 shows the average training times for a single dataset (in seconds, with a log scale on the Y-axis) for each device and each combination of SNPs and patients. The graph shows clearly that, as SNPs or patients double, the training time also roughly doubles.

#### Partitions and combinations scalability

This section focuses on assessing the impact of partitions and combinations in the model’s training time. Because increasing partitions and combination sizes also increases the network’s size, this study is done on a single node of LUMI supercomputer, since AMD MI250X GPUs have the most memory (128 GB) when compared to the other devices, and, therefore, can fit bigger models in memory. Table 1 provides the average training times for a dataset for a given set of partitions and combination size in one LUMI node (4 dual-GPUs).

**Table 1:** Average training time in seconds on a transformer with *M* partitions and *C* combinations of partitions in LUMI.

*M*	*C*							
	2	3	4	5	6	7	8	9
3	4.347	X	X	X	X	X	X	X
4	5.877	5.328	X	X	X	X	X	X
5	8.247	8.811	6.162	X	X	X	X	X
6	16.409	15.622	13.518	7.375	X	X	X	X
7	14.189	27.085	29.091	21.317	8.111	X	X	X
8	17.881	42.400	55.690	48.413	26.325	10.136	X	X
9	24.743	61.224	106.361	111.023	75.208	34.122	10.272	X
10	28.756	86.943	199.678	250.125	230.307	133.796	56.133	14.023

#### Comparison with exhaustive search

The optimal approach in terms of accuracy to identify epistatic interactions is to perform an exhaustive search across all SNP combinations, albeit at the cost of infeasible execution times. As efficient exhaustive methods for fifth order interactions and beyond do not exist, the average execution times must be extrapolated from existing works in the literature. The most relevant work for this analysis (Epi4Tensor)^10^ presents not only an efficient implementation of fourth order epistasis on GPUs, but also provides performance metrics. For 8 NVIDIA A100 GPUs, the proposed implementation achieves a performance of 835.366 evaluated tera sets ($10^{12}$) per second scaled by the sample size in a best-case scenario. According to the implementation details, the performance for higher order interactions can be extrapolated. In addition to the increase in the amount of SNP combinations to evaluate, there is also the increase in processing cost per SNP combination to account for. The approach described in^10^ relies on a dataset representation and computation method that calculates all $3^K$ genotypes from $2^K$ genotypes for an interaction order *K*, and infers the remaining genotypes from auxiliary data. Because $2^K$ values are necessary for a single SNP combination, an increase in interaction order (e.g., from fourth to fifth) is expected to increase the cost in memory and the cost of evaluating a SNP combination by a factor of 2x, while reducing performance (i.e., the evaluated tera sets per second) by the same factor. By relying on this observation it is possible to extrapolate execution times, that grow exponentially, for exhaustive search on interaction orders beyond fourth.

**Figure 3:**
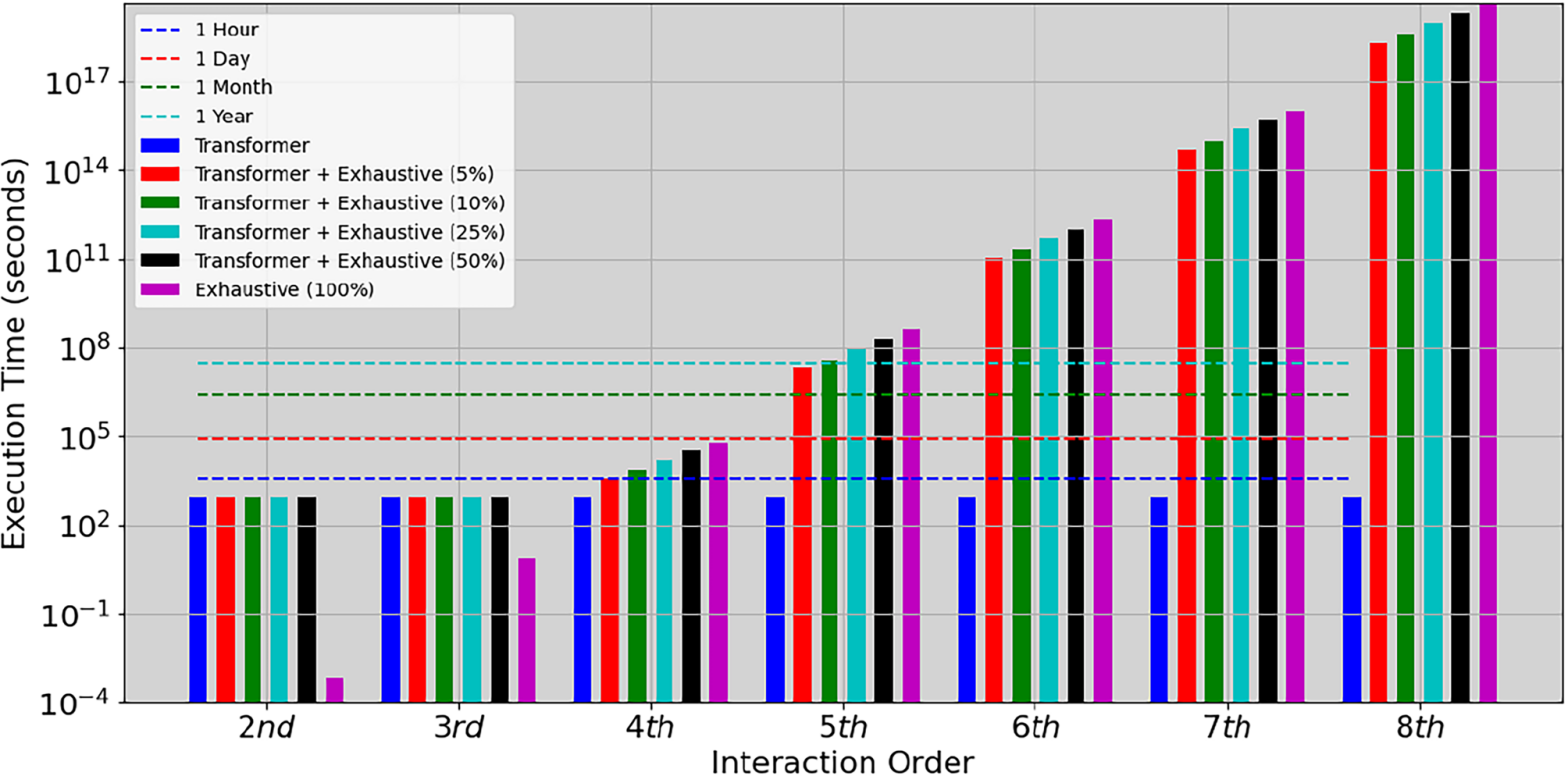
Computational time comparison between an exhaustive approach and the proposed transformer approach for a dataset with 16,384 SNPs and 16,384 samples.

Figure 3 provides the execution times for a dataset of 16,384 SNPs and 16,384 samples using the transformer model, the exhaustive search approach, and combinations of both approaches for second to eighth order interactions. The results show that employing the transformer as a first step to reduce the number of SNPs to analyze in an exhaustive search reduces the execution time for high order epistasis detection. As an example, for forth, fifth, sixth, seventh, and eighth interaction orders, employing the transformer to obtain the top 5% SNPs for exhaustive search leads to a time reduction of 16 h 20 m, 12.2 years, $6.67 \times 10^4$ years, $3.12 \times 10^8$ years, and $1.27 \times 10^{12}$ years respectively, when compared to doing a full exhaustive run.

### Evaluation on real datasets

To inspect how trustworthy the proposed framework is on a real-world scenario, experiments on WTCCC^27^ datasets for rheumatoid arthritis (RA), coronary artery disease (CAD), and inflammatory bowel disease (IBD) are performed. These three diseases are analyzed, because CAD is one of the leading causes of death in the developed world^28^, RA is a prevalent disease in older people^29^, and IBD has a significant prevalence worldwide^30^. Furthermore, these diseases are well documented in the literature in regards to genetic correlations, which is useful for validation of the proposed approach. The chosen datasets have 3476, 3448, 3453 samples, respectively, with 1500 controls and 1976, 1948 and 1953 cases, respectively. These datasets are obtained using the Affymetrix 500K SNP chip, meaning that 500,000 SNPs are covered for each sample. These SNPs are partitioned into their respective chromosomes in Table 2, which shows, for each chromosome, how many SNPs are sequenced.

**Table 2:** Number of SNPs per chromosome.

Chromosome	$\#$SNPs	Chromosome	$\#$SNPs	Chromosome	$\#$SNPs
1	40,220	9	22,864	17	11,281
2	41,400	10	28,501	18	14,881
3	33,801	11	26,273	19	6399
4	32,334	12	24,954	20	12,400
5	32,056	13	19,188	21	7125
6	31,470	14	15,721	22	6207
7	25,835	15	14,356	X	10,536
8	27,457	16	15,309		

For validation on these datasets, the developed framework runs on individual chromosomes and reports the top 1000 SNPs with highest scaled element-wise sum of attentions scores and gradients for each chromosome. For comparison, the considered state-of-the-art works (DeepCOMBI, CNN, and transformer) are also tested in these datasets. Unlike the simulated datasets, the interacting SNPs are not known in real datasets. A first approach to validate the proposed transformer’s predictions and interpretation is to find works on the studied diseases that report genes related to the disease and epistatic relationships. Afterwards, the objective is to find SNPs that are in the sets of 1000 SNPs that the proposed framework outputs and are mapped to the target genes. By searching the reported SNPs in the National Center for Biotechnology Information (NCBI) database, it is possible to determine to which genes these SNPs are mapped, as well as to calculate the physical distance in base-pairs to the gene’s starting point^31^ (see Table S3 in Supplementary Material). Table 3 provides some genes that are known to be related to the studied diseases and are related to SNPs within the 1000 SNP sets that the transformer model reports for each chromosome.

**Table 3:** Validation of identified SNPs on state-of-the-art works.

Disease	Chromosome	Gene	MLP (DeepCOMBI)	CNN	Transformer	Our approach	
			SNP (position)	SNP (position)	SNP (position)	SNP (position)	Work
RA	21	RUNX1	rs16992357 (88),	rs2268295 (139),	rs2014300 (503),	rs16992357 (88), rs7280071 (125), rs4817695 (352)	^32^
			rs2014300 (176),	rs764967 (308),	rs4817695 (808),	rs8130963 (412), rs9980210 (437), rs12482247 (461)	
			rs9976946 (271)	rs2834688 (561),	rs16992357 (946),	rs2154450 (524), rs8126925 (602), rs2834694 (681)	
				rs7281724 (676)	rs2834643 (987)	rs2834643 (731), rs2834695 (737)	
						rs762164 (883), rs2834700 (919)	
	2	STAT4	None	None	rs16833260 (574),	rs1454755 (543), rs13395651 (637),	^33^
					rs12463658 (596)	rs1356381 (831)	
	16	WFDC1	None	None	rs12447081(499)	rs7498901 (225), rs2326206 (477)	^34^
	16	CDH13	None	None	None	rs16960645 (275), rs16961631 (692), rs254346 (909)	^34^
	1	PTPN22	None	None	None	rs2488457 (23)	^34^
	6	HLA-DRA	None	None	rs1051336 (250)	rs7194 (530), rs9268645 (598)	^35^
	6	HLA-DQA1	None	None	None	rs9272346 (724), rs9272723 (942)	^35^
CAD	1	BRINP3	None	None	None	rs12724000 (1), rs1540513 (2), rs1442569 (4)	^36^
		(FAM5C))				rs12091129 (6), rs980180 (7), rs4845237 (8)	
						rs655598 (9), rs16832305 (12), rs10920713 (13)	
						rs1171048 (14), rs505600 (15), rs652953 (17)	
						rs10800939 (18), rs6691910 (19), rs725106 (21)	
						rs1148613 (23), rs12084962 (25), rs1171386 (26)	
						rs10920711 (28), rs12082492 (31)	
						rs17377331 (32), rs501982 (34)	
	4	LDB2	None	None		rs6813028 (1), rs16893733 (6), rs9291648 (25)	^37^
						rs1848040 (20), rs17429735 (692), rs16893829 (12)	
					rs1496742 (994)	rs2645255 (657), rs283027 (685), rs284208 (671)	
						rs207684 (699), rs157611 (712)	
						rs150260 (726), rs287961 (829)	
	13	COL4A1	None	None	None	rs7998488 (356)	^38^
	13	COL4A2	None	rs2281972 (114),	rs7996686 (664),	rs4773160 (319), rs4773148 (321)	^38^
				rs4773148 (133)	rs9555699 (910)	rs4773175 (359), rs4771684 (363)	
	15	SMAD3	None	rs2033785 (758)	None	rs16950559 (392), rs2053295 (535)	^38^
						rs2033784 (769), rs7174445 (945)	
IBD	2	ATG16L1	None	None		rs6752107 (383), rs3828309 (680)	^39^
					None	rs10210302 (681), rs6431654 (682)	
						rs3792106 (685), rs6737398 (998)	
	5	ERAP2	None	None	None	rs2549797 (842), rs2548533 (845)	^40^
						rs11135484 (914), rs1056893 (941)	
						rs13167902 (958), rs2549794 (967)	
	10	DLG5	None	None	rs1248631 (862)	rs10824583(838), rs7895188 (907)	^41^
	16	NOD2	None	rs17312836 (122)	None	rs17312836 (932)	^42^
	9	TLR4	None	None	rs7045953 (431)	rs1927912 (193)	^42^

For each gene, the chromosome it belongs to is identified, as well as the disease it is related to and a work in the state-of-the-art that studies its relationship to the disease. After identifying the gene and the chromosome, the set of 1000 SNPs that the transformer reports for each chromosome is analyzed to find SNPs related to the studied genes, as well as their positions in that 1000 SNP set. As Table 3 shows, the proposed framework reports several SNPs related to the evaluated genes, showing that the literature validates the obtained results.

## Discussion

The hyperparameter values to test the transformer on the simulated datasets were chosen, given that they provide the best detection power across the considered models (additive, multiplicative, threshold, and xor) and interaction orders (eighth order, except in multiplicative, which was fifth order). In general, the chosen values for partitions and combination size result in a trade-off. If the partitions are too big, the influence of noisy SNPs, when partition combinations are performed, may decrease the detection power; if the partitions are too small, there may not be enough SNPs in the partitions for the model to learn meaningful interactions. Furthermore, some sets of partitions and combination sizes yield similar topologies for the transformer model (for example, 3 partitions with 2 combinations and 6 partitions with 4 combinations). For the simulated datasets, with 1000 SNPs, the sweet spot for the parameters is 6 partitions with a combination size of 2, given the aforementioned reasons. While Table 1 shows that it is possible to run models up to 10 partitions with various combination sizes, the training times for a single dataset are a limiting factor for certain combination sizes when the number of partitions exceeds 8. These results also limited the hyperparameter search space (for example, 10 partitions with a combination size of 5 requires 250 s for a single dataset, and testing the transformer for the considered epistasis models in the hyperparameter search space would amount to 18 days of computation in a single node of the AMD MI250X supercomputer).

The results across multiple synthetic epistasis models show clearly that the proposed framework manages to outperform every other state-of-the-art interpretation-based neural network for epistasis detection for any model and interaction order up to eighth order, which, to the best of our knowledge, has never been tackled before in the literature. While the detection power in some cases is very high (over 90%), the transformer still fails in some cases, namely when the heritability is very low (0.01). Heritability is directly correlated with detection power, as it represents the proportion of people that have a given disease due to genetic factors. For example, a heritability of 0.01 means that, in 100 cases, 1 can be explained through the SNPs. As in these experiments the datasets have 800 cases (half of 1600 samples), it is most likely that the transformer does not have enough data to find the interacting SNPs. Additionally, some models have a penetrance table structure that favors certain MAF values. For example, the threshold and xor models favor high and low MAF values, respectively, and the transformer performs worse in threshold models when the MAF is low and in xor models when the MAF is high.

The analysis of interpretability power for epistasis is of the utmost importance to cross-compare different DNN architectures. Other metrics, such as time and memory requirements, would not yield straightforward comparisons, due to the different computational efforts, number of parameters, and number of layers, that each of the explored DNN architectures (MLP, CNN, and transformer) requires. The network’s training stage further increases this task’s difficulty: the different number of epochs to train each model (500, 1000, and 50 for the compared MLP, CNN, and transformer works, respectively) has a direct impact on execution time and the training’s forward, backward, and optimizer steps have different memory usages^43^ that cannot easily be estimated. These metrics, if measured for all DNNs, would show the trade-off between model complexity and accuracy. Simpler architectures, such as MLPs and CNNs, may be faster and require less memory, albeit at the cost of being unable to provide the necessary explainability for high-order epistasis, which more complex networks, such as the proposed transformer-based approach, can achieve.

While the experiments are done in datasets with 1000 SNPs, real datasets may be composed of hundreds of thousands of SNPs. Therefore, the model is trained on a plethora of AI devices to analyze performance and scalability with the input datasets, while showcasing the portability of the developed framework. To extract the most performance of the tested hardware, the batch size used for training in each case is equal to the number of available samples, which allows for each sample to be trained in parallel. However, this is done only as long as there is enough memory (e.g., for the biggest dataset, 16,384 SNPs with 16,384 samples, the transformer model runs with a batch of 4 in IPU GC-200 and a batch of 128 in TPU V4).

Regarding the results of Fig. 2, for small datasets and intermediate datasets (256–2048 SNPs), IPU GC-200 demonstrates the best performance, but as the number of samples increases, it eventually loses to TPU V4 (which holds the best times for larger datasets). It is worth mentioning that, while IPU GC-200 and TPU V4 achieve better training times than GPUs, their memory could be a limitation to their scalability, not only on the datasets, but also on the model (e.g., it may not be possible to train a model with several partitions and combinations in these devices). Testing the model for several partitions and combination sizes is done on LUMI, as AMD MI250X GPUs have the largest memory (128 GB) out of all tested devices. On that same device, as a reference, for the largest dataset (16,384 SNPs, 16,384 samples), the proposed framework is on par with CNN for execution time and on par with the transformer work for memory requirements (in terms of number of parameters).

For the experiments on WTCCC^44^ datasets, the SNP interactions are unknown. The results reported in Table 3 are SNPs related to genes for which the literature confirms susceptibility to the studied diseases. According to the physical distance calculations between SNPs and genes (see Table S3 in Supplementary Material), all reported SNPs are intragenic (i.e., they are within well-known gene boundaries, which may contribute to disease susceptibility^45^) , except rs2488457, which is a variant in the upstream promoter region of the PTPN22 gene, but is nonetheless well documented in the literature as a variant associated to RA^46^. Regarding individual correlations to the diseases, the transformer-based approach reports SNPs for RUNX1, STAT4, HLA-DRA, and HLA-DQA1, related to RA, and SNPs for ATG16L1, ERAP2, and DLG5 for IBD. The most notable results are for CAD, where the transformer is able to identify over 20 SNPS related to the BRINP3 (FAM5C) gene and 13 SNPs for the LDB2 gene, both of which have individual correlation to CAD, and for RA, as only 2 SNPs for HLA-DQA1 exist in the WTCCC dataset and the transformer is able to identify both within the selected top SNPs. When compared to the other state-of-the-art methods (MLP (DeepCOMBI), CNN, and transformer), none of the aforementioned methods is able to identify as many SNPs as the proposed transformer-based framework, even in the cases where the correspondent gene has many SNPs in the dataset (such as BRINP3 and LBD2).

Furthermore, the transformer also reports SNPs related to genes that are known to be epistatic in the literature. For CAD, the obtained results include SNPs related to SMAD3 and COL4A1/COL4A2 genes, which have been reported to have an epistatic effect on CAD^38^. For RA, an epistatic interaction between PTPN22, WFDC1, and CDH13 has been reported in the state-of-the-art^34^. Finally, for IBD, NOD2 and TLR4 have also been confirmed as being epistatic^42^. Some of the SNPs reported in the literature are not present in the WTCCC dataset, so it is not possible to fully replicate the results, but nonetheless the transformer is still able to find a relationship between these genes. Additionally, not all the evaluated genes have the same number of SNPs in the WTCCC dataset, which explains the asymmetries in the reported number of SNPs for each gene. Finally, note that the other state-of-the-art methods are not able to find these epistatic interactions, as they fail to output SNPs related to at least one of the genes in each considered interaction, demonstrating the higher reliability of the herein proposed approach.

## Methods

### Transformer model

**Figure 4:**
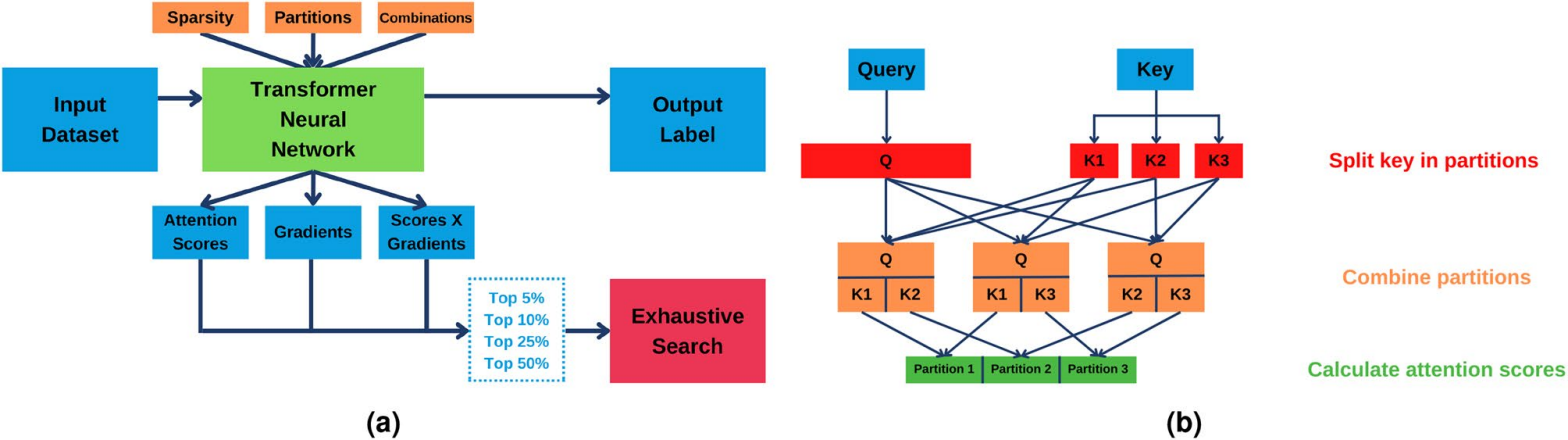
Proposed transformer-based approach for high order epistasis. (**a**) Depiction of the framework to detect SNP interactions. (**b**) Example of a partitioning scheme for the key matrix and subsequent combination of partitions.

A high level overview of the proposed transformer-based framework is provided in Fig. 4. The transformer is trained on an input epistasis dataset and outputs a label (0 if the transformer classifies a patient as a control, based on the SNPs, 1 if it classifies the patient as a case). Additionally, the transformer also outputs the values of attention scores combined with gradients for each SNPs, which are sorted afterwards. The top scoring SNPs (e.g., 5%, 10%, 25%, and 50%) are then selected for exhaustive search to identify epistatic interactions of any order. The metrics to evaluate the SNPs are configurable (e.g., attention scores or gradients can be used individually to measure a SNP’s relevance for the prediction). The transformer network also has configurable parameters, namely how sparse the weight matrices are on the attention module, as well as the number of SNP partitions and the combination order between partitions.

In previous works, a transformer-based framework^18^ is proposed for epistasis detection by leveraging the network’s capabilities for sequence modeling. In Natural Language Processing (NLP), sequence modeling refers to a task whose goal is to learn a probability distribution over a single token, *x*[*t*], using the preceding tokens, $x[1:t-1]$ as a context, Likewise, in epistasis detection, the goal is to predict the patient’s phenotype, based on the patient’s SNPs, which serve as a context. An encoder-only architecture is enough for this particular task^18^, since encoders calculate relationships between tokens.

While the method proposed herein also relies on an encoder-only transformer for epistasis detection, it diverges in comparison to previous works in two aspects: how the attention is calculated between SNPs and the phenotype and how the interpretation is done.

### Attention calculation

Attention represents the core mechanism for a transformer to predict tokens in a sentence by means of contextual information. The standard transformer employs this mechanism for sequence-to-sequence tasks, of which sequence translation is an example, to calculate attention all-to-all tokens - known simply as self-attention. In^18^, an alternate solution is considered to repurpose the transformer for epistasis detection. Given that the main objective is to predict whether a patient has a disease or not, given the SNPs (considered contextual information), a strategy known as single-query attention^47^ is proposed.

In single-query attention, the current label to predict (represented as a dense vector known as an embedding) is mapped to a query vector (*Q*) according to a trainable linear projection. Likewise, the SNPs (represented by their embeddings) are mapped to a key vector (*Y*) and a value vector (*V*). Attention is calculated as

1 $$\begin{aligned} Attention(Q,Y,V) = \underbrace{softmax\Bigg (\frac{QY^T}{\sqrt{D}}\Bigg )}_{\text {Attention Score}}V. \end{aligned}$$

where *D* is the embedding size. The inner product $QY^T$ is a measure of a SNP’s importance to predict the current label. Similar embeddings to represent a SNP *t* and a label are mapped to similar queries and keys. As a consequence, $Q{Y_t}^T$ should have a large value. Conversely, different embeddings lead to a small product, denoting a non-existent relationship between the SNP and the label. In Eq. (1), the softmax function is given by

2 $$\begin{aligned} Softmax(QY_{t}^T /\sqrt{D}) = \frac{exp(QY_{t}^T/\sqrt{D})}{\sum _t exp(QY_{t}^T/\sqrt{D})}, \end{aligned}$$

where *exp*(.) denotes the exponential function. Applying softmax to $QY^T/\sqrt{D}$ outputs a probability distribution over the SNPs, known as attention scores, which are used to combine the value vectors. As interacting SNPs should have a large $Q{Y_t}^T/\sqrt{D}$ value, the corresponding attention score should also be large. Therefore, keeping the SNPs with the highest attention scores after training provides a method to identify potential epistatic interactions. An exhaustive search can be performed afterwards on the chosen SNPs to find the optimal SNP combination.

While this approach works, it has some drawbacks. In epistatic datasets, it is unlikely that many SNPs have a true correlation to the label. Therefore, calculating attention simultaneously between all SNPs and the label may hinder the identification of epistatic interactions if most SNPs are noisy. To overcome this problem and boost the transformer’s prediction power, a possible solution is to split the key vector (which represents the SNPs) into several partitions, $Y_i$, and calculate attention between the query and a partition (the query cannot be split because it represents a single token, the patient’s label). As each partition has a smaller number of SNPs, noise is reduced, increasing the chances of identifying true epistatic SNPs. However, there is no guarantee that a single partition holds all possible interacting SNPs. Therefore, attention should be calculated between combinations of partitions, allowing for all possible subsets of SNPs to be evaluated together.

Figure 4 provides an example of this strategy. In this example, the key vector is split in three partitions and mixed in combinations of two, resulting in three different options (1 and 2, 1 and 3, 2 and 3). Attention scores are obtained for each combination and separated according to the partition they belong to. Because each partition occurs more than once in the combinations, several attention scores are obtained for the same partition, which are summed together.

### Gradient calculation

The rationale for dividing the key vector in partitions is based on the assumption that, when attention is calculated on a small subset of SNPs, interacting SNPs, if present, should be easier to identify through the attention scores. However, it may happen that certain partition combinations exist where only noisy SNPs are present, meaning the network will try to learn patterns from data that has no correlation to the output. This may influence the attention scores for each SNP and possibly reduce the transformer’s power to detect SNP interactions. To overcome this risk, an additional strategy for interpretation is considered. A previous work in the literature^48^ proposes Attentive Class Activation Tokens (Attentive CAT) for transformer explainability. CAT is defined as

3 $$\begin{aligned} {CAT_i}^L = \nabla {h_i}^L \odot {h_i}^L, \end{aligned}$$

where $\odot$ represents the Hadamard product (element-wise product), ${h_i}^L$ is the output of the i-th token from the last Transformer layer, *L*, and $\nabla {h_i}^L$ is given by

4 $$\begin{aligned} \nabla {h_i}^L = \frac{\partial y^c}{\partial {h_i}^L}, \end{aligned}$$

where $y^c$ is the transformer’s final output for class *c*. Therefore, $\nabla {h_i}^L$ illustrates a partial linearization from ${h_i}^L$ that captures the importance of the i-th token to a target class *c*. Attentive CAT is then calculated as

5 $$\begin{aligned} {AttCAT_i}^L = ({\alpha _i}^L \cdot {CAT_i}^L)_H, \end{aligned}$$

where ${\alpha _i}^L$ denotes the attention scores of the i-th token at the *L*-th layer. This result is averaged over the attention heads, *H*.

For the proposed framework, only one transformer layer exists, with a single encoder. After training, $\nabla {h_i}^L$ is calculated for each SNP between the transformer’s final output and the encoder’s output, as well as attention scores. While Attentive CAT suggests a element-wise multiplication between attention scores and gradients, for the proposed framework, element-wise sum is also calculated. Furthermore, for these calculations, both gradients and attention scores are scaled from 0 to 1, to mitigate differences in the order of magnitude of both metrics.

For element-wise sum and multiplication, averaging along the attention heads is not necessary, as the proposed network architecture works with a single attention head. In addition to these two metrics, both attention scores and gradients can also be employed separately, adding to the framework’s flexible configuration. A hyperparameter search is done to analyze the optimal network parameters, as well as which of these four interpretation metrics provides the best detection power.

### Software and hardware

The transformer model is implemented and trained using TensorFlow. Depending on the used hardware, different TensorFlow versions are employed. Most of the experiments are devised on the LUMI supercomputer, on nodes with 8 AMD MI250X GPUs (TensorFlow 2.11, 128 GB memory). For scalability and comparison purposes, the model is also trained on systems with Intel PVC (TensorFlow 2.12, 48 GB memory), NVIDIA A100 (TensorFlow 2.12, 80 GB memory), Google TPU V4 (TensorFlow 2.12, 32 GB memory) and GraphCore IPU GC-200 (TensorFlow 2.6.3, 900 MB memory).

### Dataset generation

Synthetic datasets are generated using the PyToxo^20^ and GAMETES^49^ software. PyToxo creates penetrance tables, which describe, for a given allele combination, the probability of expressing a phenotype under study. To create penetrance tables, PyToxo is provided with the epistasis model (e.g., additive with second order interactions), as well as the MAF and heritability values, to create a table that satisfies these parameters. Afterwards, GAMETES receives a penetrance table and generates datasets with interacting SNP combinations that satisfy the values of the given table. The chosen values for MAF (0.05, 0.1, 0.2, 0.5), heritability (0.01, 0.05, 0.2, 0.4), SNPs (1000) and samples (1600) are adopted from the state-of-the-art MACOED^21^.

### Hyperparameter search

Table 4 describes the analyzed possibilities for each hyperparameter for the transformer-based approach. Given the number of hyperparameters to analyze, grid search is adopted to analyze all hyperparameter combinations. However, because of the sheer number of simulated datasets to train the transformer, evaluating a single set of hyperparameters amounts to a significant computational time, because each set results in a different network size for the transformer model. To reduce this search time, the transformer is trained on the datasets with highest interaction order (eighth order for additive, threshold, and xor models, fifth order for multiplicative) to optimize the network for high order epistasis detection. During the grid search, the interpretation metrics considered in Table 4 are calculated and compared for each set of partitions and combination size.

**Table 4:** Hyperparameter search space.

Hyperparameter	Range
Partitions	3, 4, 5, 6, 7, 8
Combinations	2, 3, 4, 5, 6, 7
Interpretation metrics	Attention scores, gradients
	Scaled gradients*scores
	Scaled gradients+scores

The proposed model is trained for 15 epochs, using a 90–10 train-test split on the dataset, and Adam optimizer. The batch size is equal to the number of samples in a dataset to allow training with every sample in parallel and extract the most performance from the tested accelerators, as long as the devices do not run out of memory.

### Supplementary Information

Supplementary Information.

## Data Availability

The source code of this work is available on: https://github.com/hiperbio/episdet-transformer.
