# Supplementary material for: Distributed transformer for high order epistasis detection in large-scale datasets
Source: Sci Rep. 2024 Jun 25;14:14579. doi: 10.1038/s41598-024-65317-5 (PMC11199512; doi:10.1038/s41598-024-65317-5)
Supplement: Supplementary file 1 — Supplementary Information. [file 41598_2024_65317_MOESM1_ESM.pdf]

Supplementary Material

Hyperparameter results

Hyperparameter tuning is performed using datasets with highest interaction order for the considered epistasis models (additive, multiplicative, threshold, and xor models) and differents sets of partition and combinations values. Table 1 describes, for each set of partitions and combinations, the detection power according to the evaluated interpretation metrics (attention scores, gradients, element-wise sum of attention scores and gradients, and element-wise multiplication of attention scores and gradients). The values in bold represent the highest detection power for each model. It is possible to conclude that using 6 partitions with combinations of size 2 provides the best detection power when scaled element-wise sum of gradients and attention scores are used.

Table 1. Hyperparameter Results.

#Partitions, #Combinations	Add_8	Mul_5	Thr_8	Xor_8
(3,2)	(0.686, 0.368, 0.666, 0.521)	(0.272, 0.049, 0.268, 0.261)	(0.000, 0.010, 0.000, 0.000)	(0.000, 0.000, 0.000, 0.000)
(4,2)	(0.778, 0.232, 0.757, 0.615)	(0.382, 0.027, 0.297, 0.267)	(0.050, 0.028, 0.001, 0.000)	(0.000, 0.062, 0.000, 0.000)
(5,2)	(0.672, 0.079, 0.673, 0.564)	(0.450, 0.248, 0.500, 0.525)	(0.098, 0.031, 0.088, 0.049)	(0.004, 0.000, 0.000, 0.000)
(6,2)	(0.876, 0.490, **0.915**, 0.812)	(0.780, 0.259, **0.790**, 0.747)	(0.674, 0.003, **0.677**, 0.543)	(0.393, 0.000, **0.395**, 0.321)
(7,2)	(0.625, 0.028, 0.561, 0.312)	(0.412, 0.131, 0.378, 0.444)	(0.018, 0.015, 0.039, 0.062)	(0.000, 0.008, 0.044, 0.048)
(8,2)	(0.699, 0.471, 0.713, 0.633)	(0.532, 0.109, 0.508, 0.384)	(0.216, 0.094, 0.197, 0.178)	(0.125, 0.001, 0.111, 0.111)
(4,3)	(0.862, 0.271, 0.858, 0.749)	(0.567, 0.245, 0.573, 0.432)	(0.503, 0.088, 0.500, 0.455)	(0.249, 0.000, 0.233, 0.210)
(5,3)	(0.866, 0.379, 0.863, 0.826)	(0.655, 0.378, 0.640, 0.569)	(0.629, 0.048, 0.614, 0.559)	(0.326, 0.000, 0.290, 0.211)
(6,3)	(0.875, 0.642, 0.900, 0.751)	(0.518, 0.157, 0.468, 0.287)	(0.216, 0.112, 0.203, 0.206)	(0.108, 0.000, 0.101, 0.085)
(7,3)	(0.876, 0.424, 0.874, 0.863)	(0.725, 0.360, 0.711, 0.628)	(0.626, 0.006, 0.609, 0.505)	(0.359, 0.000, 0.338, 0.228)
(8,3)	(0.838, 0.318, 0.843, 0.716)	(0.729, 0.345, 0.721, 0.611)	(0.577, 0.003, 0.563, 0.544)	(0.343, 0.000, 0.294, 0.179)
(5,4)	(0.867, 0.361, 0.864, 0.640)	(0.725, 0.295, 0.709, 0.475)	(0.610, 0.072, 0.596, 0.545)	(0.276, 0.000, 0.263, 0.219)
(6,4)	(0.903, 0.774, 0.905, 0.834)	(0.527, 0.231, 0.506, 0.512)	(0.139, 0.128, 0.158, 0.129)	(0.117, 0.000, 0.116, 0.022)
(7,4)	(0.868, 0.403, 0.868, 0.860)	(0.656, 0.371, 0.644, 0.580)	(0.653, 0.086, 0.629, 0.579)	(0.322, 0.000, 0.306, 0.289)
(8,4)	(0.861, 0.241, 0.857, 0.825)	(0.750, 0.396, 0.745, 0.608)	(0.654, 0.080, 0.645, 0.484)	(0.368, 0.000, 0.356, 0.256)
(6,5)	(0.882, 0.679, 0.880, 0.774)	(0.537, 0.294, 0.542, 0.528)	(0.228, 0.025, 0.225, 0.166)	(0.128, 0.000, 0.122, 0.086)
(7,5)	(0.868, 0.350, 0.869, 0.769)	(0.727, 0.395, 0.717, 0.632)	(0.588, 0.034, 0.575, 0.531)	(0.284, 0.000, 0.264, 0.202)
(8,5)	(0.906, 0.431, 0.904, 0.869)	(0.741, 0.431, 0.730, 0.647)	(0.656, 0.008, 0.634, 0.555)	(0.296, 0.000, 0.263, 0.178)
(7,6)	(0.870, 0.383, 0.871, 0.792)	(0.690, 0.404, 0.696, 0.661)	(0.613, 0.128, 0.636, 0.556)	(0.191, 0.000, 0.188, 0.133)
(8,6)	(0.869, 0.319, 0.868, 0.804)	(0.659, 0.377, 0.625, 0.587)	(0.609, 0.039, 0.594, 0.543)	(0.341, 0.000, 0.326, 0.245)
(8,7)	(0.870, 0.370, 0.869, 0.757)	(0.716, 0.347, 0.707, 0.611)	(0.608, 0.080, 0.602, 0.596)	(0.180, 0.000, 0.153, 0.074)

Recall, Precision, and F1-Score

Following the procedure of MACOED, recall, precision, and F1-score are presented for all DNN approaches in Table 2. For each epistasis model, a triplet is provided for each DNN with the values for recall, precision, and F1-score, respectively. The values in bold are the best for a given epistasis model. The results show that, on average, the proposed approach achieves higher recall, precision, and F1-score values than other state-of-the-art DNN approaches for epistasis interpretation. For the four models (additive, multiplicative, threshold, and xor), on average, the proposed approach achieves 86.3%, 61.6%, 48.1%, and 19.6% in recall; 95%, 82.1%, 75.4%, and 43.4% in precision; and 90.3%, 70.3%, 58.6%, and 26.8% in F1-score. The increase over the second best method (transformer with attention scores only for additive and multiplicative models, and DeepCOMBI for threshold and xor) is 39.5%, 33.5%, 32%, and 17.7% for recall; 29%, 40.2%, 28.6%, and 33.8% for precision; and 36.9%, 31%, 41.6%, 23.6% for F1-score. The average increase in recall, precision, and F1-score is 30.6%, 26.9%, and 33.3%, respectively.

Table 2. Average recall, precision, and F1-score values for all DNN approaches across different epistasis models.

	CNN	DeepCOMBI	Transformer	Our Approach
Add_2	(0.2463,0.5378,0.3379)	(0.3680,0.6571,0.4717)	(0.3034,0.5949,0.4019)	(**0.8609,0.9771,0.9153**)
Add_3	(0.2356,0.5189,0.3241)	(0.2055,0.4846,0.2886)	(0.5537,0.7956,0.6530)	(**0.6922,0.8818,0.7756**)
Add_4	(0.4425,0.7080,0.5446)	(0.3321,0.6172,0.4318)	(0.7621,0.9374,0.8407)	(**0.8330,0.9520,0.8886**)
Add_5	(0.3224,0.6071,0.4212)	(0.4648,0.7286,0.5676)	(0.8950,**1.0000**,0.9446)	(**0.9510**,0.9979,**0.9738**)
Add_6	(0.2946,0.5800,0.3908)	(0.3566,0.6379,0.4574)	(0.6833,0.8829,0.7704)	(**0.9776,0.9986,0.9880**)
Add_7	(0.4072,0.6821,0.5100)	(0.1854,0.4600,0.2643)	(0.0474,0.2300,0.0786)	(**0.9526,0.9986,0.9751**)
Add_8	(0.2581,0.5247,0.3460)	(0.3120,0.5767,0.4049)	(0.0300,0.1773,0.0513)	(**0.7729,0.8447,0.8072**)
Mul_2	(0.1612,0.4197,0.2329)	(0.0550,0.2454,0.0898)	(0.2609,0.5335,0.3504)	(**0.4860,0.6952,0.5720**)
Mul_3	(0.1547,0.4674,0.2325)	(0.1735,0.5118,0.2591)	(0.2360,0.5960,0.3382)	(**0.6565,0.8216,0.7298**)
Mul_4	(0.0152,0.1674,0.0279)	(0.1337,0.4988,0.2109)	(0.3535,0.8107,0.4923)	(**0.6144,0.8702,0.7203**)
Mul_5	(0.0000,0.0000,0.0000)	(0.0706,0.4200,0.1208)	(0.2727,0.6975,0.3921)	(**0.7071,0.8950,0.7900**)
Thr_2	(0.1775,0.2813,0.2176)	(0.2968,0.5752,0.3916)	(0.1295,0.3798,0.1931)	(**0.5785,0.7860,0.6665**)
Thr_3	(0.0911,0.1671,0.1179)	(0.3175,0.6813,0.4331)	(0.0828,0.3479,0.1338)	(**0.5624,0.8235,0.6684**)
Thr_4	(0.1331,0.3370,0.1908)	(0.2200,0.5930,0.3209)	(0.1702,0.5220,0.2567)	(**0.4790,0.7450,0.5831**)
Thr_5	(0.0075,0.0233,0.0113)	(0.1116,0.4411,0.1781)	(0.4444,0.8889,0.5926)	(**0.3394,0.6356,0.4425**)
Thr_6	(0.0102,0.0356,0.0158)	(0.0652,0.3378,0.1093)	(0.0004,0.0278,0.0009)	(**0.4321,0.7311,0.5432**)
Thr_7	(0.0044,0.0156,0.0068)	(0.0621,0.3322,0.1047)	(0.0023,0.0644,0.0045)	(**0.4619,0.7922,0.5835**)
Thr_8	(0.0000,0.0000,0.0000)	(0.0557,0.3144,0.0946)	(0.0039,0.0833,0.0075)	(**0.5168,0.7633,0.6163**)
Xor_2	(0.0420,0.2050,0.0698)	(0.0819,0.2863,0.1273)	(0.0702,0.2650,0.1110)	(**0.3468,0.5888,0.4365**)
Xor_3	(0.0052,0.0719,0.0097)	(0.0376,0.1938,0.0630)	(0.0188,0.1369,0.0330)	(**0.2621,0.5119,0.3467**)
Xor_4	(0.0007,0.0269,0.0014)	(0.0125,0.1119,0.0225)	(0.0100,0.1000,0.0182)	(**0.1885,0.4344,0.2629**)
Xor_5	(0.0025,0.0500,0.0048)	(0.0003,0.0163,0.0005)	(0.0043,0.0656,0.0081)	(**0.1839,0.4288,0.2574**)
Xor_6	(0.0000,0.0019,0.0000)	(0.0043,0.0656,0.0081)	(0.0000,0.0019,0.0000)	(**0.1421,0.3769,0.2064**)
Xor_7	(0.0000,0.0000,0.0000)	(0.0000,0.0000,0.0000)	(0.0000,0.0013,0.0000)	(**0.0930,0.3050,0.1426**)
Xor_8	(0.0000,0.0000,0.0000)	(0.0000,0.0000,0.0000)	(0.0000,0.0000,0.0000)	(**0.1560,0.3950,0.2237**)

Genomic Distance

In Table 3, the initial and final positions of each gene are provided within the chromosome that the gene belongs to. The genomic distance between a SNP and its corresponding gene is calculated using the gene's initial position as a reference. SNPs are ordered according to this distance for each gene, which is also provided in the table, along with the SNP's position in the chromosome. This information was retrieved from the NCBI database. The results show that all genes are intragenic (their positions are within a known gene's boundaries) to the exception of rs2488457 (PTPN22) which is in a regulatory DNA section.

Table 3. Gene and SNP positions and distance in base-pairs (bp) from each reported SNP to the gene it belongs to.

Gene (positions)	SNP (position, distance in bp)
RUNX1 (21:34787801-36004667)	rs16992357 (21:34803153, 15352), rs2834643 (21:34814099, 26298), rs9976946 (21:34835765, 47964) rs7280071 (21:34836726, 48925), rs4817695 (21:34847020, 59219), rs2154450 (21:34847269, 59468) rs762164 (21:34846477, 59676), rs2268295 (21:34871435, 83634), rs8126925 (21:34872939, 85138) rs8130963 (21:34895121, 107320), rs764967 (21:34927368, 139567), rs2834688 (21:34946566, 158765) rs2834694 (21:34963684, 175883), rs2834695 (21:34963726, 175925), rs2834700 (21:34965294, 177493) rs2014300 (21:34985564, 197763), rs7281724 (21:34993923, 206122) rs12482247 (21:35033168, 245367), rs9980210 (21:35034253, 246452)
STAT4 (2:16501541-16898678)	rs1356381 (2:191059687, 30111), rs12463658 (2:191100618, 71042), rs16833260 (2:191106839, 77263) rs1454755 (2:191209477, 179901), rs13395651 (2:191209803, 180227)
WFDC1 (16:84294846-84329844)	rs2326206 (16:84306286, 11440), rs12447081 (16:84307409, 12563), rs7498901 (16:84318725, 23879)
CDH13 (16:82626965-83800640)	rs16960645 (16:83486693, 859728), rs16961631 (16:83747999, 1121034), rs254346 (16:83749345, 1122380)
PTPN22 (1:113813811-113871753)	rs2488457 (1:113872746, 58935)
HLA-DRA (6:32439878-32445046)	rs9268645 (6:32440750, 872), rs7194 (6:32444703, 4825), rs1051336 (6:32444815, 4937)
HLA-DQA1 (6:32628179-32647062)	rs9272346 (6:32636595, 8416), rs9272723 (6:32641650, 13471)
BRINP3 (1:190097658-190478404)	rs10800939 (1:190273120, 175462), rs505600 (1:190311829, 214171), rs655598 (1:190318583, 220925) rs652953 (1:190319167, 221509), rs501982 (1:190321190, 223532), rs1540513 (1:190332402, 234744) rs1171386 (1:190343195, 245537), rs980180 (1:190347978, 250320), rs1442569 (1:190353597, 255939) rs12084962 (1:190357498, 259840), rs6691910 (1:190368023, 270365), rs1148613 (1:190368170, 270512) rs725106 (1:190378535, 280877), rs16832305 (1:190382026, 284368) ,s12724000 (1:190382547, 284889) rs10920711 (1:190392324, 294666), rs17377331 (1:190400378, 302720), rs10920713 (1:190400839, 303181) rs4845237 (1:190413665, 316007), rs12082492 (1:190415508, 317850) rs12091129 (1:190437201, 339543), rs1171048 (1:190474186, 376528)
LDB2 (4:16501541-16898678)	rs1496742 (4:16607054, 105513), rs1848040 (4:16619766, 118225), rs16893733 (4:16670171, 168630) rs9291648 (4:16682663, 181122), rs16893829 (4:16697898, 196357), rs2645255 (4:16724583, 223042) rs6813028 (4:16731133, 229592), rs17429735 (4:16731847, 230306), rs283027 (4:16752240, 250699) rs284208 (4:16754302, 252761), rs207684 (4:16771596, 270055), rs150260 (4:16775552, 274011) rs157611 (4:16779557, 278016), rs287961 (4:16788692, 287151)
SMAD3 (15:67063763-67195173)	rs16950559 (15:67085470, 21707), rs2053295 (15:67099401, 35638), rs2033785 (15:67149528, 85765) rs2033784 (15:67157322, 93559), rs7174445 (15:67158877, 95114)
COL4A1 (13:110148963-110307202)	rs7998488 (13:110155588, 6625)
COL4A2 (13:110305812-110513209)	rs4773148 (13:110311081, 5269), rs4773160 (13:110342413, 36601), rs4773175 (13:110394225, 88413) rs7996686 (13:110440890, 135078), rs9555699 (13:110447671, 141859) rs2281972 (13:110478397, 172585), rs4771684 (13:110504426, 198614)
ATG16L1 (2:233210051-233295674)	rs10210302 (2:233250193, 40142), rs6752107 (2:233252802, 42751), rs6431654 (2:233253123, 43072) rs6737398 (2:233261751, 51700), rs3828309 (2:233271764, 61713), rs3792106 (2:233282094, 72043)
ERAP2 (5:96875986-96919703)	rs11135484 (5:96886185, 10199), rs13167902 (5:96886316, 10330), rs2548533 (5:96902697, 26711) rs2549794 (5:96908845, 32859), rs1056893 (5:96909735, 33749), rs2549797 (5:96909814, 33828)
DLG5 (10:77790791-77926755)	rs1248631 (10:77823123, 32332), rs10824583 (10:77859644, 68853) rs7895188 (10:77863620, 72829)
NOD2 (16:50693588-50733077)	rs17312836 (16:50707551, 13963)
TLR4 (9:117704175-117724735)	rs1927912 (9:117707148, 2973), rs7045953 (9:117723517, 19342)
